# Clinical Data for Parametrization of *In Silico* Bone Models Incorporating Cell-Cytokine Dynamics: A Systematic Review of Literature

**DOI:** 10.3389/fbioe.2022.901720

**Published:** 2022-07-12

**Authors:** Charles Ledoux, Daniele Boaretti, Akanksha Sachan, Ralph Müller, Caitlyn J. Collins

**Affiliations:** ^1^ Institute for Biomechanics, ETH Zurich, Zurich, Switzerland; ^2^ Department of Chemical Engineering, Indian Institute of Technology Bombay, Mumbai, India; ^3^ Department for Biomedical Engineering and Mechanics, Virginia Tech, Blacksburg, VI, United States

**Keywords:** bone, osteoporosis, aging, agent - based modeling, cell population dynamics, cytokine, parametrization approach, verification and validation

## Abstract

*In silico* simulations aim to provide fast, inexpensive, and ethical alternatives to years of costly experimentation on animals and humans for studying bone remodeling, its deregulation during osteoporosis and the effect of therapeutics. Within the varied spectrum of *in silico* modeling techniques, bone cell population dynamics and agent-based multiphysics simulations have recently emerged as useful tools to simulate the effect of specific signaling pathways. In these models, parameters for cell and cytokine behavior are set based on experimental values found in literature; however, their use is currently limited by the lack of clinical *in vivo* data on cell numbers and their behavior as well as cytokine concentrations, diffusion, decay and reaction rates. Further, the settings used for these parameters vary across research groups, prohibiting effective cross-comparisons. This review summarizes and evaluates the clinical trial literature that can serve as input or validation for *in silico* models of bone remodeling incorporating cells and cytokine dynamics in post-menopausal women in treatment, and control scenarios. The GRADE system was used to determine the level of confidence in the reported data, and areas lacking in reported measures such as binding site occupancy, reaction rates and cell proliferation, differentiation and apoptosis rates were highlighted as targets for further research. We propose a consensus for the range of values that can be used for the cell and cytokine settings related to the RANKL-RANK-OPG, TGF-β and sclerostin pathways and a Levels of Evidence-based method to estimate parameters missing from clinical trial literature.

## 1 Introduction

Global life expectancy increased from 66.8 years in 2000 to 73.4 years in 2019 (Cao and Ho, 2020). This has led to a rise in the prevalence of osteoporosis (OP), which is associated with an increased risk of bone fracture. An estimated 250,000 deaths occurred in the European Union, Switzerland and the United Kingdom in 2019 as a direct result of hip or spine fragility fractures (Kanis et al., 2021); however, only approximately 25% of individuals at high risk for fracture receive pharmacologic therapies other than supplemental calcium and vitamin D (McCloskey et al., 2021). Given the high incidence of both the disease, related fractures, and patient morbidity and mortality, current diagnostic and treatment approaches appear to be insufficient for the healthcare needs of the world’s aging population. This is despite the wide spectrum of approved drugs for OP treatment, whose use has been shown to reduce the incidence of hip fracture in clinical trials by 40% (Black et al., 1996; Ettinger, 1999; Watts et al., 2003; Black et al., 2006; Black et al., 2012; Miller et al., 2012; Inderjeeth et al., 2014; Takakuwa and Iwamoto, 2015; Lello et al., 2017). The treatment gap is caused in part by the high cost of some therapeutic options (Suhm et al., 2008), which is in turn linked to the long and large clinical trials required to observe statistically significant changes in bone quality and fracture risk and obtain drug approval (May 2019).

Traditionally, research efforts to address these issues have focused on creating *in vitro* and preclinical models to add to the screening and clinical trial design steps in the drug discovery pipeline. However, the majority of new drugs fail in the animal model stage or fail to translate to effective treatment for humans, leading to a significant loss in time and financial resources. *In silico* simulations have been proposed to provide fast, inexpensive and ethical alternatives to years of costly experimentation on animals and humans (Webster, 2020; Nikolova-Simons et al., 2021; Sarrami-Foroushani et al., 2021). Existing *in silico* models have been developed to study bone remodeling, its deregulation during metabolic bone diseases and the effect of therapeutics and exercise (Lemaire et al., 2004; Pivonka et al., 2008; Lerebours et al., 2015; Pastrama et al., 2018; Boaretti et al., 2019; Tourolle, 2019; Kameo et al., 2020; Martínez-Reina et al., 2021a; Tourolle et al., 2021).

Bone remodeling is the local repair and adaptation of the mineralized collagen matrix within bone tissue by osteoblasts, bone forming cells, and osteoclasts, bone resorbing cells. This process is coordinated by osteocytes, mechanically sensitive cells located in interconnected cavities within the bone matrix. The balance between resorption and formation is a delicate equilibrium regulated by various cell-cytokine pathways including but not limited to: estrogen-induced or -inhibited apoptosis (Khosla et al., 2012); osteoclastogenesis regulated by receptor activator of nuclear factor *?* β (RANK)-its ligand (RANKL) and Osteoprotegerin (OPG) (Boyce and Xing, 2008); mechanosensitivity via the Wingless and int-1 (Wnt) proteins, sclerostin and parathyroid hormone (PTH) (Jilka et al., 1999; Hadjidakis and Androulakis, 2006; Lewiecki, 2014; Kovács et al., 2019); osteoblastogenesis governed by levels of bone morphogenetic protein (BMP) and transforming growth factor-β (TGF-β) (Hanada et al., 1997; Zimmermann et al., 2005; Giannoudis et al., 2008); and interleukin-governed interplay between bone remodeling and immune reactions (Shaarawy et al., 2003; Pino et al., 2010; Sun et al., 2011; Giganti et al., 2012; Marques et al., 2013). Within the varied spectrum of *in silico* modeling techniques, only bone cell population dynamics models (Lemaire et al., 2004; Pivonka et al., 2008; Lerebours et al., 2015; Pastrama et al., 2018; Martínez-Reina et al., 2021a) and micro-multiphysics agent-based (micro-MPA) models (Boaretti et al., 2019; Tourolle, 2019; Kameo et al., 2020; Tourolle et al., 2021) explicitly incorporate the complex cellular and molecular mechanisms causing metabolic bone diseases and the pathways involved in their treatments.

Bone cell population dynamics models (Lemaire et al., 2004; Pivonka et al., 2008; Lerebours et al., 2015; Pastrama et al., 2018; Martínez-Reina et al., 2021a) divide bone into representative volume elements (RVE). Within the RVE, the model stores one concentration per cytokine and the population of every cell type. The resorption and formation activities of the cells in the RVE as well as their proliferation, differentiation and apoptosis are then determined as a function of the cytokine concentrations using Hill coefficients for repression and activation. Within each RVE, the local bone thinning or thickening is proportional to a weighted balance between the osteoclast and osteoblast numbers.

Evolving from the work of early bone cell population dynamics models, micro-MPA models of bone mechanobiology represent every cell as an independent agent that senses its local environment (i.e. cytokine concentrations and mechanical signals) and modifies it, leading to the emergence of complex patterns at the local and global level as observed in clinical patient data. To date, only micro-MPA models have the resolution to predict the effect of the complex cell-cytokine pathways in bone on the bone microarchitecture. Tourolle (Tourolle et al., 2021) and Kameo (Kameo et al., 2020) developed micro-MPA models of osteoporosis and its treatments with bisphosphonates and RANK ligand inhibitors, respectively, using microcomputed tomography (microCT) scans of iliac crest biopsies with resolutions on the order of 10 µm as input.

Despite the promise of such *in silico* models, extensive verification and validation are still needed, particularly using human data, for micro-MPA models or bone cell population dynamics models to be applied clinically. In these types of models, parameters for cell and cytokine behavior are set based on experimental values found in literature; however, the settings used vary from one research group to the next (Kameo et al., 2020; Tourolle et al., 2021) as well as from one model to another (Martínez-Reina et al., 2021a; Tourolle et al., 2021). The cell-cytokine signaling pathways included in micro-MPA models of bone remodeling were based on earlier bone cell population dynamics models. All bone cell population dynamics models and micro-MPA models of bone developed to date include the RANK-RANKL-OPG pathway and the three major bone cell types, osteoblasts, osteoclasts and osteocytes, but the choice of other cells and cytokines to include has varied across models as shown in Table 1. To ensure the longevity of micro-MPA models and bone cell population dynamics models and to facilitate their clinical translation, a common set of ground rules is required (Erdemir et al., 2020).

**TABLE 1:** Bone cell population dynamics models and micro-MPA models and the cell-cytokine pathways included in each model.

	Cytokines (Besides RANKL and OPG)					Cells (BESIDES Osteoclasts and osteoBLASTS)				
Study	PTH	TGF-**β**	SCLR	WnT	OTHER	osteocytes	pre-osteoclasts	pre-osteoblasts	lining cell	other
Komarova et al. (2003)		x			IGF		x	x		
LEMAIRE 2004 (Lemaire et al., 2004)	x	x					x	xx		
Pivonka 2008 (Pivonka et al., 2008) 2010 (Pivonka et al., 2010)	x	x					x	xx		
Buenzli 2011 (Buenzli et al., 2011) 2012 (Buenzli et al., 2012)	x	x					x	xx		
PIVONKA 2013 (Pivonka et al., 2013)	x	x			MCSF		xx	xx		
SCHeiner 2013 (Scheiner et al., 2013) 2014 (Scheiner et al., 2014)	x	x					x	xx		
Lerebours 2016 (Lerebours et al., 2015)	x	x			MCSF		xx	xx		
pastrama 2018 (Pastrama et al., 2018)	x	x	x	x		x	x	xx		
Martinez-reina 2019 (Martínez-Reina and Pivonka, 2019) 2021 (Martínez-Reina et al., 2021a)^,^ (Martínez-Reina et al., 2021b)		x				x	x	xx		
martin 2019 (Martin et al., 2019) 2020 (Martin et al., 2020)		x	x	x	NO	x	x	xx		
Lavaill 2020 (Lavaill et al., 2020)	x	x				x	x	xx		
***Kameo 2020*** (Kameo et al., 2020)			x		Sema3A	x				
***tourolle 2021*** (Tourolle et al., 2021)		x	x		estrogen	x	xx	x	x	preosteocyte

Abbreviations: Rank: Recptor Activator of Nuclear, Factor Κ Β, Rankl: Rank Ligand, OPG: Osteoprotegerin, PTH: Parathyroid Hormone, TGF- Β: Transforming Growth, Factor Β, SCLR, Sclerostin, WNT: Wingless and INT-1 Proteins, IGF, Insulin-Like Growth, Factor; MCSF, Macrophage Colony Stimulating, Factor; NO, Nitric Oxide; SEMA3A: Semaphorin, 3A. Bold: Bone Cell Population Dynamics Models, *Italics: MICRO-MPAS*, X: Indicates, parameter, was accounted for in model, XX: Indicates, parameter, was accounted for in the model using two cell types.

This review aims to evaluate and summarize clinical trial literature that can serve as input to micro-MPA models or bone cell population dynamics models of postmenopausal osteoporosis (PMO) and its treatments; thus, providing a consensus for the range of values that can be used for each cell or cytokine behavior setting. The literature will be assessed using the Grading of Recommendations, Assessment, Development and Evaluations (GRADE) system to determine the level of confidence of the reported data (Balshem et al., 2011). Areas lacking in reported cell or cytokine measures with high confidence will be highlighted as targets for further research and guidelines will be formulated to fill gaps in the clinical evidence based on an adaptation of the Oxford Center for Evidence Based Medicine (CEBM) Levels of Evidence.

## 2 Methods

### 2.1 Search Strategy

In this review, we collect and analyze data exclusively on the cell-cytokine pathways that have consistently been included in previous micro-MPA models and bone cell population dynamics models of bone, specifically osteoblasts, osteoclasts, osteocytes, RANK-RANKL-OPG, sclerostin, and TGF-β. As there is no direct correspondence between each precursor cell genotype used in micro-MPA models and bone cell population dynamics models and a specific *in vivo* cell genotype, precursors were excluded from this review. The aim of the search strategy outlined below was to identify articles containing data that could be used for the parametrization or verification of micro-MPA models of bone or bone cell population dynamics models, i.e. any peer-reviewed articles reporting measurements of parameters relating to cell and cytokine behavior in bone from postmenopausal women.

To identify scientific articles reporting measures relevant for model parametrization and verification, the researchers conducted searches of the three most widely used journal databases in the biomedical and life sciences; MEDLINE (PubMed), Scopus and Science Citation Index Expanded (Web of Science, WoS).

The search terms utilized to target relevant studies are shown in Table 2. All searches were accompanied with the keywords “postmenopausal” and “osteoporosis”, and articles with titles containing: “mice”, “rats”, “murine”, “pigs”, “*in vitro*”, “in silico”, “osteogenesis imperfecta”, “corticosteroid”, “glucocorticoid” were excluded. In PubMed, the search was restricted to “Clinical trials” and “Randomized Controlled Trial”. In Scopus the filters “Article”, “Journal” and “English” were also applied. In Web of Science, the search was restricted to articles; thus, reviews, editorial material, meeting abstracts, and proceedings papers were excluded.

**TABLE 2:** Search terms and number of resultant quantitative reports on experimental measurements relating to parameters that have to date been used in agent-based models (micro-MPA models) of bone or bone cell population dynamics models.

	Search Terms (20.09.2021)	PubMed	Scopus	WoS
Cytokines	RANKL	38	21	266
	OPG	28	38	320
	Estrogen	2	18	2
	Sclerostin	42	47	6
	TGF	2	46	56
Cells	Osteoclast	2	54	8
	Osteoblast	1	14	2
	Osteocyte	7	35	47
	Preosteoclast	2	10	1
	HSC	1	2	1
	MSC	2	33	12
Techniques	Histomorphometry	39	185	170
	CT	132	578	2
	Histology	44	171	3

The results of these searches were analyzed to identify articles providing data on cell and cytokine parameters in postmenopausal women receiving any approved treatments for PMO or acting as untreated controls. Each article identified by this search process was reviewed and included in the current work if it met all three of the following criteria:

1) The article was a case-control or a cohort study published as an original article
2) The findings of the study included quantitative reports on cell numbers or cell proliferation, differentiation, cytokine production or apoptosis rates or cytokine concentrations, diffusion, decay or reaction rates
3) The article reported the above data for post-menopausal women.

A detailed list of the applied exclusion criteria may be found in Supplementary Appendix B.

### 2.2 Assessment of Quality of Evidence Using Grading of Recommendations, Assessment, Development and Evaluations Guidelines

Every article that fit the inclusion criteria was evaluated using the GRADE guidelines for the evaluation of the quality of evidence (Balshem et al., 2011) to create a trustworthiness score that could be used to explain quantitative differences in the values reported for each parameter.

Two reviewers (CL, AS) independently evaluated the evidence provided in each source for its quality using the systematic GRADE approach, which methodologically assesses studies for five characteristics: risk of bias (Guyatt et al., 2011a), inconsistency or heterogeneity of results across studies (Guyatt et al., 2011b), indirectness (Guyatt et al., 2011c), imprecision (Guyatt et al., 2011d) and publication bias (Guyatt et al., 2011e). The quality of a body of evidence for a particular outcome can be downgraded depending on the seriousness of the risk of bias; unexplained and important inconsistency; indirect study results with uncertain relation to relevant cytokine concentrations, cell numbers, etc. for postmenopausal women; small sample size that influences the width of the confidence intervals which express the uncertainty about the magnitude of the effect; and detection of publication bias. The two reviewers evaluated two articles together to coordinate their assessment strategy then proceeded to independently evaluate the same six articles to validate that the GRADE score assigned to each article was identical. The remaining articles were split among the two reviewers.

Note the GRADE guidelines were designed and are used as a tool to assess the quality of evidence for a drug in the context of therapeutic decision-making. In the context of assessing the quality of evidence for reported parameter values, the five main evaluation criteria (i.e. risk of bias, inconsistency, indirectness, imprecision and publication bias) remained the same in the current work; however, individual subcriteria were interpreted in a manner that differs slightly from the traditional approach. The specific subcriteria that were interpreted differently are outlined below:

Within the risk of bias category, the subcriterion *incomplete outcome data* was evaluated for randomized trials as the *difference between the number of patients enrolled and the number of patients for which the parameter of interest was reported*. In cases where there were unexplained differences, studies were downgraded based on this subcriterion.

Within the inconsistency category, the subcriterion *probability that subgroup differences may be attributed to chance* was refined. Here for the parametrization of cell numbers and cytokine concentrations, evidence was downgraded if different patient demographics displayed overlapping error bars and upgraded if the difference between different patient subgroups was highly significant (*p*-value < 0.01 set to further stratify the search results). When measures across subgroups were expected to be similar (e.g. gene polymorphisms), the statistical insignificance of differences between the means was used as an upgrade criterion since it indicated repeatability of the measurement.

Within the imprecision category, the quality of evidence was upgraded for *sample size* if there were more than 100 serum measurements of cytokines or more than 10 measurements of cytokines in bone marrow plasma or cell numbers in biopsies. As before, these thresholds were chosen to further stratify the search results.

All other criteria were assessed as outlined in detail elsewhere (Guyatt et al., 2011a; Guyatt et al., 2011b; Guyatt et al., 2011c; Guyatt et al., 2011d; Guyatt et al., 2011e) and a GRADE score for the quality of the evidence was determined out of 6 for each measurement.

### 2.3 Data Features

**FIGURE 1:**
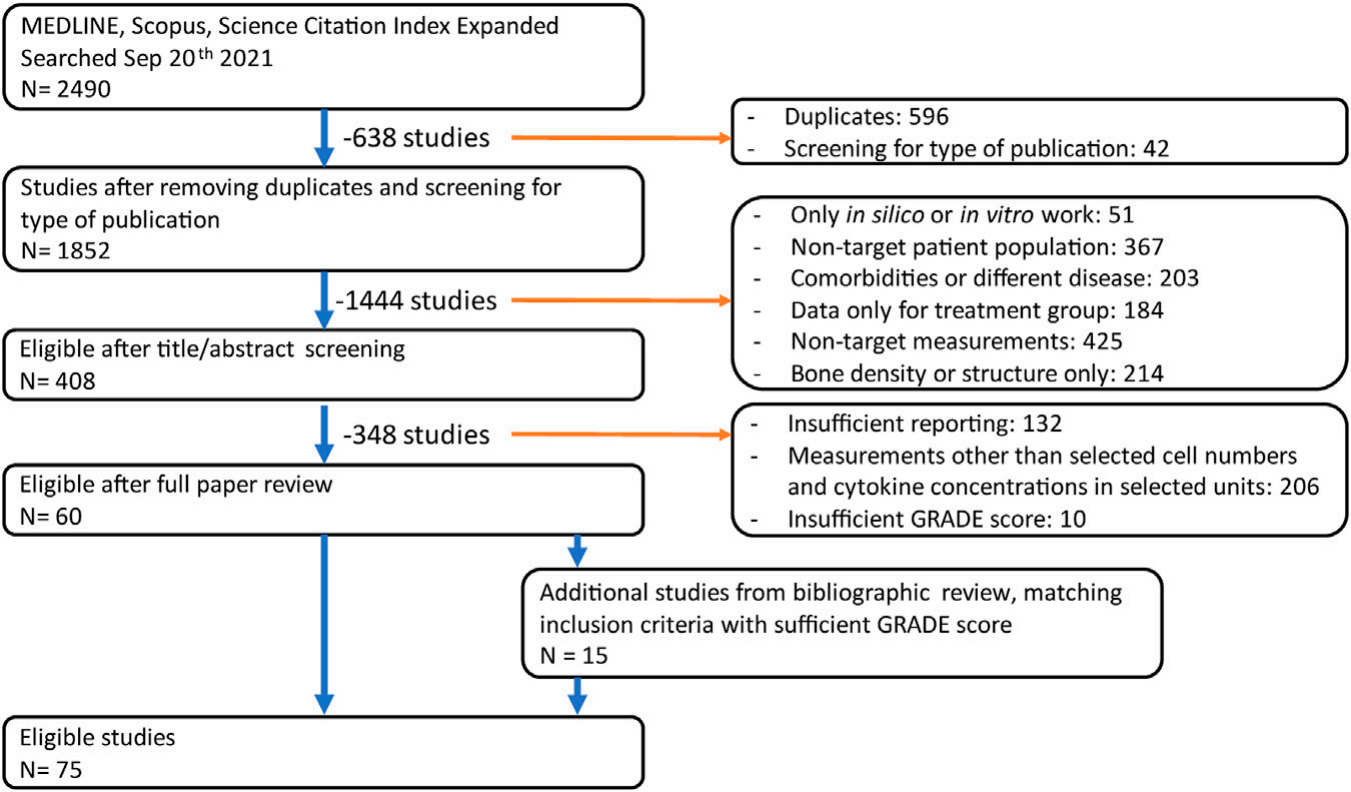
PRISMA diagram illustrating the effect of each inclusion/exclusion criterion.

The searches in the databases PubMed, Scopus and WoS yielded a total of 1852 non-duplicate results suitable for analysis. Based on the title and abstracts only, 408 unique peer-reviewed articles in the databases PubMed, Scopus and WoS fit the inclusion criteria and were evaluated for relevance considering the full text. Then, quantitative measures relating to cytokine or cell numbers in postmenopausal women were identified and the quality of the evidence was evaluated using the GRADE guidelines. This reduced the total to 60 articles containing quantitative reports on cell and cytokine behavior in postmenopausal women. Finally, a bibliographic search was conducted on these 60 articles and an additional 17 articles were included based on full article review and GRADE analysis, as illustrated in Figure 1.

### 2.4 Methodology for Analysis of Physiologic Ranges for Cell and Cytokine Parameters

This review collects, where available from patient data, parameters such as cell numbers, cytokine concentrations, cytokine reaction rates, cell proliferation and apoptosis rates, cell motility, the effects of cytokines on cell behavior and the production rates of cytokines by cells. For cases where patient data is unavailable, cell and cytokine settings are supplemented with values from pre-clinical animal studies, *in vitro* work or computational studies.

For all cytokines that have to date been included in micro-MPA models of bone or bone cell population dynamics models, differences in the average concentrations reported in various papers were compared with differences in study design. Factors including measurement technique, the origin of the sample or location of the measurement in the body, characteristics of the patient population and the quality of the evidence as assessed using the GRADE guidelines were considered to explain differences in reported values. To facilitate comparison between cytokine concentrations reported in different units, mass concentrations were converted to molar concentrations using molar masses of 20.00 kDa for free RANKL, 19.90 kDa for OPG, 0.27 kDa for estrogen, 22.5 kDa for sclerostin and 4.76 kDa for TGF-β, following the recommendations of Biomedica, Vienna, Austria, the manufacturer of the most widely used human RANKL and OPG assays (as formulated in the instructions for use of ELISA kits BI-20403, BI-20462 and BI-20492).

Similar to the cytokine concentration analysis, differences in study design were accounted for prior to comparison of the reported average cell number for each of the cell number parameters included in micro-MPA models of bone or bone cell population dynamics models (Table 1). There is no consensus regarding conversion between cell surface and cell number therefore articles that assess osteoblast surface (Ob. S/BS, %) and osteoclast surface (Oc. S/BS, %) rather than number were not included in this review (Mullender et al., 2005). As an added challenge, cell numbers across studies are often reported in different units, and there is no standardized way to infer cell numbers in mm^−1^, mm^−2^ and mm^−3^ from each other (Parfitt et al., 1987). In this review, we used two inference methods to qualitatively compare cell numbers reported in different units.

Osteoclast and osteoblast numbers were all measured in histology sections from iliac crest biopsies and reported in units of either cells/mm or cells/mm^2^. Histology sections are typically analyzed with light microscopes coupled with specific analysis software (e.g., OsteoMeasure™, OsteoMetrics, Decatur, GA, United States). Cell numbers reported by this commercial software in cells/mm of surface are obtained by counting the number of osteoclasts or osteoblasts in a histological section and dividing it by the length of the interface between bone and marrow. We propose to divide these cell numbers in cells/mm by the product of the section thickness and a factor of 2.23 to account for curvature using the parallel surface method (Nishikawa et al., 1998; Jinnai et al., 2002). The resulting number must then be divided by the ratio of the average cell diameter to the section thickness to account for the fact that cells larger than the section thickness would be counted repeatedly if the section were displaced to its nearest neighboring location in the direction normal to the cutting plane. So in effect, the proposed inference method involves dividing the cell number in cells/mm by 2.23 times the cell diameter to estimate the cell number in cells/mm^2^. Whenever the cell diameter was not provided, a reference of 150 µm was used for osteoclasts and 40 µm for osteoblasts (Manolagas, 2000).

Osteocyte numbers are measured either using histology sections observed with a microscope and reported in cells/mm^2^ or using µCT imaging of bone biopsies and reported in cells/mm^3^. Our proposed inference method in this review involves dividing the osteocyte number by the section thickness to obtain the number of osteocytes per unit of volume of the histology section then adjusting for the fact that osteocytes are larger than the section thickness by multiplying by the ratio of section thickness to average osteocyte diameter, thus in effect dividing the osteocyte number in cells/mm^2^ by the long axis length of osteocyte lacunae (by default, 8 µm (Hannah et al., 2010)) to infer osteocyte numbers in cells/mm^3^. The surface density of osteoclasts and osteoblasts in cells/mm^2^ and the volumetric dispersion of osteocytes in cells/mm^3^ may directly be compared to the corresponding parameters in the 3-dimensional finite element matrix in agent-based models of bone.

Contradictory evidence in literature was reconciled by examining all factors of the study design that could contribute to the observed differences, including the number of postmenopausal women who participated, their average age, their BMD bracket at baseline and the GRADE score evaluating the level of confidence in the study design and execution.

Care must be taken when interpreting reported values as methodological differences in measuring the relevant cell numbers and cytokine concentrations exist between imaging methods, even when manufactured by the same supplier.

## 3 Results & Discussion

An overview of the parameters related to cell and cytokine signaling pathways that have to date been included in micro-MPA models of bone remodeling or bone cell population dynamics models is presented in Table 3. The table highlights the challenging reconciliation of the differences between values reported in various studies that often span several orders of magnitude.

**TABLE 3:** Average values reported in the literature for bone cell numbers and cytokine concentrations in postmenopausal women, including all anatomical sites, imaging techniques and study designs.

Parameter	Unit	Average value reported
C_RANKL_	pg/ml	1.95 (Chiba et al., 2009); 3.8 (Dobnig et al., 2006); 5.5 (Abrahamsen et al., 2005); 8.5 (Kim et al., 2007); (8.6^OP^ 8.8^non−OP^) (Mezquita-Raya et al., 2005); (34.0^OP^ 42.6 ^non−OP^) (Azizieh et al., 2019); (208 ^non−OP^ 240 ^OP^) (Anastasilakis et al., 2008a); (1,900^non−OP^ 2,300^OP^) (Zhao et al., 2016; Coulson et al., 2017); (2,490^non−OP^ 2,880^OP^) (Molnár et al., 2014); 14,600 (D’Amelio et al., 2010a)
	pM	0.04 (Jørgensen et al., 2009); 0.07 (Gossiel et al., 2016); 0.10 (Jørgensen et al., 2011); 0.1 (LaCroix et al., 2013); (0.14^OP^ 0.27^non−OP^) (Pino et al., 2010) 0.31 (Rahnama et al., 2013) 0.505 (Anastasilakis et al., 2008b); (0.37^non−OP^ 0.66^OP^) (Jabbar et al., 2011); 1.23 (Li et al., 2011); 155.4 (Dozio et al., 2020); 4,203 (Mödder et al., 2011); 8,600 (Poornima et al., 2014)
C_OPG_	pg/ml	68.4 (Chiba et al., 2009); 83.7 (Dobnig et al., 2006); (111^OP^ 127^non−OP^) (Mezquita-Raya et al., 2005); (191^non−OP^ 488^OP^) (Wanby et al., 2016); (466.5^OP^ 471.3^OPE^ 563.3^non−OPE^) (Azizieh et al., 2019); (797^OP^ 814^OPE^) (Anastasilakis et al., 2008a); 1,359 (Oh et al., 2004); (1,300^non−OP^ 1,700^OP^) (Zhao et al., 2016; Coulson et al., 2017); 3,521 (Jørgensen et al., 2009); 9,300 (Kim et al., 2007)
	pM	1.8 (Abrahamsen et al., 2005); 3.32 (Messalli et al., 2007); 3.7 (Poornima et al., 2014); 4.35 (Wu et al., 2010); 5.0 (LaCroix et al., 2013); 5.3 (Indridason et al., 2005); 5.8 (Samelson et al., 2008); 6.27 (Rahnama et al., 2013); 6.94 (Li et al., 2011); 6.96 (Jiang et al., 2008); 7.5 (Dozio et al., 2020); 11.6 (Karadag-Saygi et al., 2011); 12.3 (Rogers et al., 2002); (10.44^non−OP^ 18.70^OP^) (Jabbar et al., 2011); 15.22 (Anastasilakis et al., 2008b); 37.5 (D’Amelio et al., 2010b); 68.2 (Mashavi et al., 2017)
C_estrogen_	pg/ml	(18.3^OP^ 21.6^non−OP^) (Mezquita-Raya et al., 2005) 23.7 (Ahlborg et al., 2003); 55 (Orwoll et al., 1989); 60.7 (Slemenda et al., 1987); 71.7 (Ouyang et al., 1984)
	pM	11.1–122.7 (Mödder et al., 2011); 27.5 (Dick et al., 2005); 27.9 (Devine et al., 2004); 38.9 (García-Martín et al., 2011); 61.0 (Rogers et al., 2002); 90–120 (Slemenda et al., 1987); 151.4 (Wu et al., 2010); (156.5^OP^ 172.1^non−OP^) (Jabbar et al., 2011)
C_sclerostin_	pg/ml	1,020 (Peng et al., 2021); 2,200 (Wanby et al., 2016)
	pM	10.44 (Sarahrudi et al., 2012); 26.7 (Clarke and Drake, 2013); 55.8 (Ardawi et al., 2012); 80.0 (Mödder et al., 2011); 192.9 (Coulson et al., 2017)
C_TGF-β_	pg/ml	(119.4^non−OPE^ 157.6^OPE^) (Azizieh et al., 2019); 5,500 (Grainger, 1999); (15,800^OP^ 23,800^non−OP^) (Faraji et al., 2016); 23,870 (Lau et al., 2004); 27,940 (Djurovic et al., 2000); 38,900 (Wu et al., 2010)^,^ (Xie et al., 2013); 40,700 (Hinke et al., 2001); 41,000 (Hinke et al., 2001); 56,700 (Tsourdi et al., 2019)
	pM	8.8 (Pfeilschifter et al., 1998)
N.Ob	mm^−1^	0.59 (Hodsman et al., 2000); 1.0 (Gruber et al., 2000); 9.3 ^Tb,^ (Jähn-Rickert et al., 2020)
	mm^−2^	0.67 (Ott et al., 2009); 6.8 ^Tb,^ (Gruber et al., 1986)
N.Oc	mm^−1^	0.02 ^OP,^ (Arlot et al., 1990); 0.022 ^OP,^ (Chavassieux et al., 2019); 0.0244 (Rehman et al., 1994); 0.05 ^OP,^ (Weinstein et al., 2009); 0.057 ^OPE,^ (Chavassieux et al., 1997); 0.07- ^OP,^ (Gruber et al., 2000); (0.03^non−OP^ 0.14^OP^) (Arlot et al., 1990); 0.13^OP,^ (Hodsman et al., 2000); 0.22 (Jobke et al., 2014); (0.364^Ct^ 0.396^Tb^) (Dekker et al., 2018)
	mm^−2^	(0.034^Ct^ 0.064^Tb^) (Rehman et al., 1994); 0.09^137^; 0.1^138^; (0.22^OP^ 0.28^non−OP^) (Carasco et al., 1989); 0.65^OP,^ (Ott et al., 2009); 0.96^OP,^ (Gruber et al., 1986); 2.35 (Cohen-Solal et al., 1995)
N.Ot	mm^−2^	148^Tb,^ (Qiu et al., 2010); (137.6^OP^ 158.5^non−OP^) (Rolvien et al., 2020); (125^OP^ 188^non−OP^) (Qiu et al., 2003); (222.6^OP^ 271.3^non−OP^) (Mullender et al., 2005); 247 ^Ct,OP,^ (Milovanovic et al., 2014)
	mm^−3^	(17,402^Ct^ 20,850^Tb^) (Akhter et al., 2017); 20,573 ^Ct,^ (Dong et al., 2014)

^non−OP^, only non-osteoporotic; ^OP^, only osteoporotic; ^OPE^, only osteopenic; ^Tb^ only trabecular; ^Ct^ only cortical. Definitions of OP, and OPE, varied across studies, all studies used a definition based on t-scores below -2.5 and -1, respectively but the location varied and the number of prior fractures was used as an additional criterion for OP, in some cases.

### 3.1 Cytokine Concentrations

In total, 56 peer-reviewed articles reporting measurements of RANKL (C_RANKL_, *n* = 21), OPG (C_OPG_, *n* = 28), estrogen (C_estrogen_, *n* = 12), sclerostin (C_sclerostin_, *n* = 7) and TGF-β (C_TGF-β_, *n* = 10) concentrations in post-menopausal women achieved a sufficient GRADE score for further analyses; the corresponding average concentrations are listed in Tables 4–8, respectively. To assist in initialization and define physiologic ranges for each concentration, T-score inclusion criteria, average patient age, size of the patient population (N), origin of the sample and GRADE score were reported. Additionally, assay characteristics and inter-assay coefficient of variation (CV) were reported for C_RANKL_ (Table 4), C_OPG_ (Table 5), C_estrogren_ (Table 6), C_sclerostin_ (Table 7), and C_TGF-β_ (Table 8) to account for differences in measurement technique. Note that RANKL assays must be differentiated in terms of whether they assess free soluble RANKL (FREE), the monomer with molecular weight 20 kDa, or total soluble RANKL (TOTAL), including both RANKL monomer and the RANKL-OPG trimer with molecular weight 60 kDa. As such, the nature of C_RANKL_ was reported when possible (Table 4).

**TABLE 4:** RANKL concentration measurements in postmenopausal women, in ascending order.

Study	C_RANKL_	BMD	Age	N	Sample origin	Assay characteristics	free/Total	Inter-assay CV	GRADE
Jørgensen 2009 (Jørgensen et al., 2009)	0.0419 pM	all	63.3	1,496	serum	Free RANKL ELISA, ampli sRANKL human, Biomedica, Vienna, Austria	Free	<5%	4
GOSSIEL 2016 (Gossiel et al., 2016)	0.07 pM	OP	65.8	62	serum	Free RANKL manual sandwich enzyme immunoassays, Biomedica, Vienna, Austria	Free	4.2%	3
CHIBA 2009 (Chiba et al., 2009)	1.95 pg/ml = 0.098 pM	OP	71.2	26	serum	sandwiched ELISA, Biomedia Co., Ltd., Nonthaburi, Thailand	Free	not available	3
Jørgensen 2011 (Jørgensen et al., 2011)	0.10 pM	All	63.4	1,596	serum	ELISA, R&D Systems, Abingdon, United Kingdom	Free	15.0%	4
LaCroix 2013 (LaCroix et al., 2013)	0.1 pM	All	69.8	400	serum	OPG ELISA kit Biomedica, Vienna, Austria	Free	6%	4
PINO 2010 (Pino et al., 2010)	0.14 pM 0.27 pM	OP non-OP	72.5 71.4	47	Iliac crest bone marrow	s-RANKL kit, Immunodiagnostic Systems, Fountain Hills, AZ, United States	Free	not available	4
DOBNIG 2006 (Dobnig et al., 2006)	3.8 pg/ml = 0.19 pM	OP	68	56	serum	polyclonal antibody-based sandwich enzyme immunoassay Biomedica, Vienna, Austria	Free	7.5%	3
ABRAHAMSEN 2005 (Abrahamsen et al., 2005)	5.5 pg/ml = 0.28 pM	all	≈52	30	serum	ELISA, Biomedica, Vienna, Austria	Free	<9%	2
KIM 2007 (Kim et al., 2007)	8.5 pg/ml = 0.43 pM	all	58	385	serum	Enzyme immunoassay kit, Biomedica, Vienna, Austria	Free	7.2%	4
MEZQUITA-RAYA 2005 (Dobnig et al., 2006)	8.6 pg/ml = 0.43 pM 8.8 pg/ml = 0.44 pM	OP non-OP	63 59	111 95	serum	sRANKL assay, sensitivity 1.6 pg/ml, Biomedica, Vienna, Austria	Free	<10%	4
RAHNAMA 2013 (Rahnama et al., 2013)	0.31 pM	all	55.4	30	serum	ELISA Anti-Human CD254 RANKL Purified, Biomedica, Vienna, Austria	Free	not available	2
JABBAR 2011 (Jabbar et al., 2011)	0.37 pM 0.66 pM	non-OP OP	62.3	370	serum	two-site sandwich ELISA, detection antibody: biotinylated polyclonal anti-RANKL ab, conjugate is streptavidine HRP conjugate and the substrate is TMB (tetramethylbenzidine) solution, Biomedica, Vienna, Austria	Free	<9%	4
ANASTASILAKIS 2008 (Anastasilakis et al., 2008b)	0.505 pM	OP	66.7	23	serum	ELISA; sensitivity 0.08 pM Biomedica, Vienna, Austria	Free	<6–9%	3
Azizieh 2019 (Azizieh et al., 2019)	34.0 pg/ml = 1.7 pM 42.6 pg/ml = 2.1 pM 42.7 pg/ml = 2.1 pM	OP non-OPE OPE	61.356.1 58.7	71	serum	HRNKLMAG-51 K MILLIPLEX MAP magnetic bead assay accuracy 92%, sensitivity 5.0 pg/ml, Merck Millipore, Darmstadt, Germany	Free	<15%	3
ANASTASILAKIS 2008 (Anastasilakis et al., 2008a)	208 pg/ml = 10.4 pM 240 pg/ml = 12.0 pM	OPE OP	64.1	74	serum	Sandwich ELISA sRANKL, sensitivity 1.6 pg/ml, PeproTech EC Ltd., London, United Kingdom	not available	<9%	3
Zhao 2016 (Zhao et al., 2016)	1900 pg/ml = 95 pM 2,300 pg/ml = 115 pM	non-OP OP	55.7 57.3	25 25	serum	ELISA kits, Apotech, Immunodiagnostic, CA, United States	not available	10%	3
Molnar 2014 (Molnár et al., 2014)	2.49 ng/ml = 125 pM 2.88 ng/ml = 144 pM	OPE OP	65	31 41	serum	sandwich ELISA, PeproTech, Cranbury, NJ, United States	not available	not available	3
DOZIO 2020 (Dozio et al., 2020)	155.4 pM	all	69.0	20	serum	sRANKL (total) human ELISA kit RD193004200R, BioVendor, Brno, Czech Republic	Total	12.7%	4
D’AMELIO 2010 (D’Amelio et al., 2010b)	14.6 ng/ml = 243 pM	OP	64	35	serum	Total sRANKL ELISA kit K1016 Apotech Corporation, Epalinges, Switzerland & Immunodiagnostik, Bensheim, Germany	Total	≤9.3%	4
Mödder 2011 (Mödder et al., 2011)	4,203 pM	non-OP	70.5	16	Iliac crest bone marrow	Total sRANKL quantitative ELISA, ALPCO, Salem, NH, United States	Total	<9.5%	4
PoOrnima 2014 (Poornima et al., 2014)	8,600 pM	all	59	291	serum	Total sRANKL ELISA, ALPCO Immunoassays, Salem, NH, United States	Total	9.3%	3

**TABLE 5:** Average OPG concentration measurements in postmenopausal women, in ascending order.

Study	C_OPG_	BMD	Age	N	Sample origin	Assay characteristics	Free/Total	Inter-assay CV	GRADE
ABRAHAMSEN 2005 (Abrahamsen et al., 2005)	1.8 pM	all	39.4	19	serum	OPG ELISA kit, Biomedica, Vienna, Austria	Free	<10%	2
PINO 2010 (Pino et al., 2010)	2.9 pM 4.4 pM	non-OP OP	71.4 72.5	8 8	Iliac crest bone marrow	OPG ELISA kit, Immunodiagnostic Systems, Fountain Hills, AZ, United States	Free	not available	4
MESSALLI 2007 (Messalli et al., 2007)	3.32 pM	all	55.3	37	serum	OPG ELISA kit, Biomedica, Vienna, Austria	Free	8.9%	3
CHIBA 2009 (Chiba et al., 2009)	68.42 pg/ml = 3.4 pM	t ≤ −2.5	71.2	26	serum	sandwiched ELISA, Biomedia Co., Ltd., Nonthaburi, Thailand	Free	not available	3
PoOrnima 2014 (Poornima et al., 2014)	3.7 pM	all	59	291	serum	ELISA, ALPCO Immunoassays, Salem, NH, United States	Free	7.6%	3
DOBNIG 2006 (Dobnig et al., 2006)	83.7 pg/ml = 4.2 pM	t ≤ −2.5	68	56	serum	polyclonal antibody-based sandwich ELISA, Biomedica, Vienna, Austria	Free	10%	3
Mödder 2011 (Mödder et al., 2011)	4.33 pM	non-OP	71.5	680	Iliac crest bone marrow	OPG quantitative enzyme immunoassay, ALPCO, Salem, NH, United States	Free	8%	4
Wu 2010 (Wu et al., 2010) Xie 2013 (Xie et al., 2013)	4.35 pM	all	60.7	269	serum	OPG ELISA kit, Biomedica, Vienna, Austria	Free	8.2%	4
LaCroix 2013 (LaCroix et al., 2013)	5 pM	all	69.8	400	serum	median OPG from ELISA kit that detects monomeric and dimeric OPG as well as OPG-RANKL complexes, Biomedica, Vienna, Austria	Total	8%	4
Indridason (Indridason et al., 2005)	5.3 pM	all	70	126	serum	OPG ELISA kit, Biomedica, Vienna, Austria	Free	4.1%	4
MEZQUITA-RAYA 2005 (Dobnig et al., 2006)	111 pg/ml = 5.58 pM 127 pg/ml = 6.38 pM	OP non-OP	63 59	111 95	serum	OPG assay, detects monomer and dimer and OPG bound to its ligand, sensitivity 3 pg/ml, Biomedica, Vienna, Austria	Total	<12%	4
samelsoN 2008 (Abrahamsen et al., 2005)	5.8 pM	all	64	1,371	serum	Total OPG ELISA kit, Biomedica, Vienna, Austria	Total	<5%	4
RAHNAMA 2013 (Rahnama et al., 2013)	6.27 pM	all	55.4	30	serum	OPG ELISA kit, Biomedica, Vienna, Austria	Free	<5%	2
JIANG 2008 (Jiang et al., 2008)	6.96 pM	OP	78.3	60	serum	OPG ELISA kit BI-20402; Biomedica, Vienna, Austria	Free	7–8%	4
Dozio 2020 (Dozio et al., 2020)	7.5 pM	OP	69	20	plasma	Human OPG ELISA kit, BioVendor Laboratory Medicine, Palackeho, Czech Republic	Total	9%	4
Wanby 2016 (Wanby et al., 2016)	191 pg/ml = 9.6 pM 488 pg/ml = 24.5 pM	non-OP OP	77 86	50 62	serum	Human Bone Magnetic Bead Panel, Merck Millipore, Burlington, MA, United States	Total	3.8%	4
JABBAR 2011 (Jabbar et al., 2011)	10.44 pM 18.70 pM	non-OP OP	62.3	370	serum	two-site sandwich ELISA, detection antibody monoclonal anti-OPG antibody, conjugate is streptavidine HRP conjugate and the substrate is TMB (tetramethylbenzidine) solution	Total	<10%	4
KARADAG-SAYGI (Karadag-Saygi et al., 2011)	11.6 pM	all	64	34	serum	polyclonal antibody-based sandwich enzyme immunoassay Immundiagnostik, Bensheim, Germany	Total	7%	2
ROGERS 2002 (Rogers et al., 2002)	12.3 pM	all	67	180	serum	sandwich ELISA, Immundiagnostik, Bensheim, Germany	Total	9.3%	5
ANASTASILAKIS 2008 (Anastasilakis et al., 2008b)	15.22 pM	OP	66.7	23	serum	ELISA; detects all three different forms of circulating OPG (monomer, dimer, and RANKL/OPG complex) RayBiotech, Peachtree Corners, GA, United States	Total	<12%	3
Oh 2004 (Oh et al., 2004)	1,358.5 pg/ml = 22.6 pM	All	54.4	137	serum	ELISA: monoclonal IgG antibody was used as a capture antibody and a biotin-labelled polyclonal antihuman OPG antibody was used as a detection antibody Oscotec, Seongnam, Korea	Total	6·0–9·0%	4
Azizieh 2019 (Azizieh et al., 2019)	466.5 pg/ml = 23.4 pM 471.3 pg/ml = 23.7 pM 563.3 pg/ml = 28.3 pM	OP OPE non-OPE	61.358.7 56.1	71	serum	HBNMAG-51 K MILLIPLEX MAP^®^ magnetic bead assay, Merck Millipore, Darmstadt, Germany	Total	<15%	3
D’AMELIO 2009 (D’Amelio et al., 2010b)	37.5 pM	OP	64	35	serum	OPG ELISA kit, Biomedica, Vienna, Austria	Total	<5%	4
ANASTASILAKIS 2008 (Anastasilakis et al., 2008a)	796.5 g/ml = 40.0 pM 814 pg/ml = 40.9 pM	OP OPE	64.1	74	serum	ELISA, RayBiotech, Peachtree Corners, GA, United States	Total	<12%	3
COULSON 2017 (Coulson et al., 2017)	1,300 pg/ml = 65.3 pM 1700 pg/ml = 85.4 pM	non-OP OP	74.0	143	serum	Multiplex immunoassay, Merck Millipore, Darmstadt, Germany	Total	not available	3
Zhao 2016 (Zhao et al., 2016)	1,300 pg/ml = 65.3 pM 1700 pg/ml = 85.4 pM	non-OP OP	55.7 57.3	25 25	serum	ELISA kits BioVendor Laboratory Medicine, Palackeho, Czech Republic	Total	7.5%	4
MASHAVI 2017 (Mashavi et al., 2017)	68.2 pM	OP	66.4	51	serum	ELISA, BioVendor Laboratory Medicine, Palackeho, Czech Republic	Total	<7.5%	3
Jørgensen 2010	3,521 pg/ml = 176.9 pM	All	63.3	1,496	serum	ELISA using mouse antihuman OPG antibody, R&D Systems, Minneapolis, MN, United States	Total	6.8%	4
KIM 2007 (Kim et al., 2007)	9.3 ng/ml = 467 pM	all	58	385	serum	Enzyme immunoassay kit, Biomedica, Vienna, Austria	Total	7.2%	4

**TABLE 6:** Estrogen concentration measurements in postmenopausal women, in ascending order.

Study	C_estrogen_	BMD	Age	N	Sample origin	Assay characteristics	E1/E2/Total	Inter-assay CV	GRADE
Mödder 2011 (Mödder et al., 2011)	6.2 pM 27.1 pM	all	71.5	16	serum	Measured E2 & E1 separately using liquid chromatography- mass spectrometry API 5000, Applied Biosystems-MDS Sciex, Framhingham, MA, United States	E2 E1	8%	4
DICK 2005 (Dick et al., 2005)	27.5 pM	all	>70	293	serum	E2 RIA, biological variation + intra-assay CV: 17.2%, Orion Diagnostica/Aidian, Espoo, Finland	E2	<7.5%	4
DEVINE 2005 (Devine et al., 2004)	27.9 pM	all	75	1,499	serum	E2 RIA, biological variation + intra-assay CV: 17.2%, Orion Diagnostica/Aidian, Espoo, Finland	E2	<7.5%	5
GARCIA-MARTIN 2011 (García-Martín et al., 2011)	38.9 pM	non-OP	56.2	92	serum	Inmunoassay. References range for PM women is < 5.0–54.7 pg/ml. Roche Elecsys 1,010/2010	Total	6.2%	2
ROGERS 2002 (Rogers et al., 2002)	61.0 pM	all	67	180	serum	Elecsys 2010 autoanalyzer total estradiol, less than 1% cross reactivity with other estrogen metabolites	Total	4.4–6.0%	5
MEZQUITA-RAYA 2005 (Dobnig et al., 2006)	18.3 pg/ml = 67.2 pM 21.6 pg/ml = 79.3 pM	OP non-OP	63 59	111 95	serum	DSL-39100 3rd Generation Estradiol RIA, Diagnostic System Laboratories, Inc., Texas	E2	<10%	4
AHLBORG 2009 (Ahlborg et al., 2003)	23.7 pg/ml = 86.8 pM	all	67	108	serum	Radioimmunoassay	E2	not available	5
Wu 2010 (Wu et al., 2010)	151.44 pM	all	60.7	269	serum	E2 RIA kit (Biotechnology Institute of the North, Beijing, China)	Total	not available	4
JABBAR 2011 (Jabbar et al., 2011)	156.46 pM 172.05 pM	OP non-OP	62.3	370	serum	electrochemiluminescence immunoassay ‘ECLIA’ implemented on the Roche Elecsys 1,010/2010 and MODULAR ANALYTICS E170 (Elecsys module) immunoassay analysers	E2	not available	4
ORWOLL 1989 (Orwoll et al., 1989)	55 pg/ml = 204 pM	OP	68.4	31	serum	I7β-estradiol assay, Wien Laboratories, Succasunna NY United States	E2	not available	2
SLEMENDA 1987 (Slemenda et al., 1987)	60.7 pg/ml = 224.8 pM	all	52.3	31	serum	E1 and E2 RIA: solvent extraction, celite chromatography for steroid purification, followed by immunoassay with specific antibodies and dextran-coated charcoal to separate free and bound steroid	Total	not available	3
OUYANG 1984 (Ouyang et al., 1984)	71.7 pg/ml = 265.6 pM	all	50	30	serum	RSL1125 total estrogen kit l Radioassay System Laboratories Carson, California, United States 3:2 mixture of ethyl acetate and hexane for extraction of serum E	Total	not available	3

**TABLE 7:** Sclerostin concentration measurements in postmenopausal women, in ascending order.

Study	C_sclerostin_	BMD	Age	N	Sample origin	Assay characteristics	Form	Inter-assay CV	GRADE
sarahrudi 2012 (Sarahrudi et al., 2012)	10.44 pM	all	63.3	1,496	serum	ELISA antibody for SOST, Biomedica	Total	<5%	4
HAMPSON 2013 (Hampson et al., 2013)	26.7 pM	all	61.6	149	serum	ELISA, Biomedica	Total	5.4%	5
Peng 2021 (Peng et al., 2021)	1,020 pg/ml = 45.3 pM	all	74.7	76	serum	ELISA (R&D Systems) specificity: natural & recombin. human SOST, sensitivity:3.8 pg/L	Total	not available	3
ARDAWI 2012 (Ardawi et al., 2012)	55.8 pM	non-OP	61	707	serum	ELISA, Biomedica	Total	6%	5
Mödder 2011 (Mödder et al., 2011)	80.0 pM	all	71.5	16	Iliac crest bone marrow plasma	ELISA, Biomedica & ALPCO	Total	4%	4
Wanby 2016 (Wanby et al., 2016)	2.2 μg/L	all	171	82	serum	Merck Millipore’s instructions for the xMAP technology with multiplex beads. Plates (Human Bone Magnetic Bead Panel from Merck Millipore) were measured using the Luminex’s xMAP^®^ instrument MagPix LX 200 (Luminex, Austin, TX, United States). All samples analyzed in duplicates	Total	3.8%	4
COULSON 2017 (Coulson et al., 2017)	192.9 pM	all	74	143	serum	Multiplex immunoassays (Millipore, Billerica, MA, United States) magnetic bead panels, sensitivity 31.1	Total	not available	3

**TABLE 8:** TGF-β concentration measurements in postmenopausal women, in ascending order.

Study	C_TGF-b_	BMD	Age	N	Sample origin	Assay characteristics	Type1/Total	Inter-assay CV	GRADE
AZIZIEH 2019 (Azizieh et al., 2019)	119.4 pg/ml = 4.77 pM 157.6 pg/ml = 6.3 pM	non-OPE OPE	56.1 59.6	25 46	serum	MILLIPLEX MAP HCYP2MAG-62 K Human Cytokine Magnetic Bead Panel, Merck Millipore, Darmstadt, Germany	Total	<15%	5
Pfeilschifter 1998 (Pfeilschifter et al., 1998)	8.8 pM	all	63.3	883	serum	ELISA, Genzyme/Sekisui Diagnostics, Burlington, MA, United States	Total	<10%	4
GRAINGER 1999 (Grainger, 1999)	5.5 ng/ml = 220 pM	all	57.7	340	serum	Active plus acid-activatable latent TGF-β BDA19 Capture ELISA, R&D Systems, Oxford, United Kingdom	type 1	not available	5
FARAJi 2016 (Faraji et al., 2016)	23.8 ng/ml = 359 pM 15.8 ng/ml = 541 pM	non-OP OP	53.4 53.7	69 65	serum	ELISA TGF-β1 kit, R&D systems, Abingdon, United Kingdom, sensitivity 15.4 pg/ml	type 1	6.1%	4
LAU 2004 (Lau et al., 2004)	23.87 ng/ml = 543 pM	OP	65.4	237	serum	ELISA, Biosource International, CA, United States	type 1	<8.9%	3
hinke 2001 (Hinke et al., 2001)	40.7 ng/ml = 926 pM	all	64.2	60	serum	ELISA using natural human TGF-β1 as a standard, Genzyme Diagnostics, Cambridge, MA, United States	type 1	not available	3
YAMADA 1998 (Yoshiji et al., 1998)	41 ng/ml = 932 pM	all	67	44	serum	ELISA kit Amersham	Total	<13.4%	2
Djurovic 2000 (Djurovic et al., 2000)	27.94 ng/ml = 1,118 pM	all	66	49	serum	ELISA-Quantikine kit, R&D Systems	type 1	9.24%	3
Wu 2010 (Wu et al., 2010) Xie 2013 (Xie et al., 2013)	38.9 ug/l = 1,556 pM	all	60.7	269	serum	ELISA kit, DRG International Inc., Highway, Mountainside, NJ, minimum detectable 0.002 μg/L	Total	8.8%	4
TSOURDI 2019 (Tsourdi et al., 2019)	56.7 ng/ml = 2,268 pM	OP	68.9	30	serum	ELISA, Immundiagnostik AG, Bensheim, Germany	Total	<14%	3

RANKL is the cytokine for which the range of average concentrations reported in peer-reviewed literature in post-menopausal women is the widest (from 0.0419 pM to 8,600 pM). From Table 4, reported RANKL measures may be divided into free RANKL ranging from 0.0419 to 2.1 pM and total RANKL ranging from 155.4 pM. to 8,600 pM; thus, tracking the differences in measurement technique reduces the parameter range from 5 orders of magnitude to 2. The type of RANKL (free vs. total) detected by a given assay was not always directly reported (Anastasilakis et al., 2008a; Molnár et al., 2014; Zhao et al., 2016), necessitating further investigation and assessment of manufacturer guidelines for individual assays. Lack of consistent reporting methods contributes to our inability to make conclusions regarding the correlation between RANKL and BMD in postmenopausal women. The study reporting the highest RANKL average concentration reported highly skewed measurements, with a median of 1,377 pM and an average of 8,600 pM^94^. Conflicting results were found regarding RANKL concentration in postmenopausal women with and without an OP diagnosis. One peer-reviewed article reported lower free RANKL concentration in postmenopausal women with OP than without (*p* = 0.021) (Pino et al., 2010), two found no significant difference (Mezquita-Raya et al., 2005; Azizieh et al., 2019) and one found higher free RANKL concentration in postmenopausal women with OP than without (*p* < 0.0001). Among the studies for which the type of RANKL detected (i.e. free vs. total) was unclear, two reported non-significant differences in average RANKL concentration between women with and without OP (Anastasilakis et al., 2008a; Zhao et al., 2016) and one reported higher RANKL concentrations in post-menopausal women with OP than in post-menopausal women with OPE (*p* < 0.027) (Molnár et al., 2014). Both the study reporting lower free RANKL in post-menopausal women with OP than without (Pino et al., 2010) and the study reporting higher free RANKL in post-menopausal women with OP than without (Jabbar et al., 2011) achieved a GRADE score of 4. so we have with this review found no evidence that free RANKL is different in the osteoporotic subgroup with respect to the non-osteoporotic subgroup. The wide range of average RANKL concentrations reported in literature may be attributed primarily to differences in measurement techniques across studies. Although a wide range of assays were used to assess RANKL concentration, only two studies reported measurements of RANKL concentration directly in bone marrow supernatant fluid. These studies both received high GRADE scores of 4. Eliminating studies with a GRADE score of 1, 2 or 3 reduces the parameter range to 0.04–0.66 pM for free RANKL and 155 pM to 4,203 pM for total RANKL.

OPG concentrations reported in literature span two orders of magnitude from 1.8 to 467 pM The type of OPG detected by a given assay was not always directly reported, necessitating further investigation and assessment of manufacturer guidelines for individual assays. There was significant overlap between the range of values reported for assays detecting the OPG monomer only and the range of values reported for assays detecting OPG in all its circulating forms, i.e. monomer, dimer and RANKL-OPG complex. Three articles comparing OPG concentrations in postmenopausal women with and without an OP diagnosis reported higher OPG concentrations in postmenopausal women with OP than without (*p* = 0.035 (Pino et al., 2010), *p* < 0.001 (Wanby et al., 2016), *p* < 0.0001 (Jabbar et al., 2011)) while three articles reported the opposite trend (*p* = 0.034 (Mezquita-Raya et al., 2005), *p* = 0.025 normal BMD vs. osteoporotic (Azizieh et al., 2019), *p* = 0.001 significance of positive association between OPG and WBMD (Coulson et al., 2017)). 12 of the 28 articles in Table 5 used OPG assays manufactured by Biomedica, Vienna, Austria, including the articles reporting the lowest OPG average concentration and the highest OPG average concentration, suggesting differences in assay characteristics cannot explain the wide range in reported values. The maximum interassay variability reported for the commercial assays used to perform these OPG concentration measurements in post-menopausal women was only 15%; thus, the interpatient variability in OPG cannot explain the wide range in the values reported for OPG either. Eliminating studies with GRADE scores of 1, 2 and/or 3 could not reduce the parameter range. Nonetheless, the GRADE score approach identified only one article with a GRADE score of 5 and this article reported an average OPG concentration of 12.3 pM which is within the allowable ranges of all high GRADE-score studies reporting *in vivo* OPG concentrations. Modelers are of course encouraged to directly measure OPG concentration levels in their own patient populations, especially to set OPG concentration levels for patient-specific work. The OPG concentration level in micro-MPA simulations or bone cell population dynamics simulations should be a target for model sensitivity testing since there are no conclusive values.

Estrogen concentrations were more consistent across studies with lower and upper bounds of 27.5 and 265.6 pM, respectively. The collected data supports a negative correlation between age and estrogen level. All reported estrogen concentrations for post-menopausal women in their fifties and sixties were higher than for post-menopausal women in their seventies. The GRADE score approach identified the articles of the highest quality. These articles reported on average values of 61.0 pM in postmenopausal women below the age of 70 and 27.5 pM in postmenopausal women above the age of 70 and these values are therefore recommended as a reference for both postmenopausal women with and without osteoporosis. Note that, as has been extensively reviewed elsewhere, for modeling purposes a question even more critical than the estrogen concentrations themselves may be the parameters relating to the effect of estrogen on bone cells as various occasionally conflicting mechanisms have been reported (Finkelman et al., 1992; Tomkinson et al., 1997; Kousteni et al., 2001; Eghbali-Fatourechi et al., 2003; Nakamura et al., 2007; Hawse et al., 2008; Pacifici, 2008; Imai et al., 2009; Martin-Millan et al., 2010).

Levels of sclerostin reported in the literature vary from 10.44 to 192.9 pM The receptor for sclerostin, LRP6, has been identified on osteoblasts, osteoclast precursors and osteocytes. Specifically, sclerostin impairs osteoblastogenesis and osteoblast survival, increases differentiation of osteoclast precursors to osteoclasts and increases production rates of sclerostin and RANKL by osteocytes (Lewiecki, 2014). The full range of concentrations reported in literature may be reduced to 10.44–80.0 pM using either the GRADE score or a differentiation based on demographics or measurement methods. The main role of sclerostin is to inhibit or stop bone formation. The data presented in Table 7 supports a positive correlation between sclerostin level and age. All reported average sclerostin concentrations in studies with average age of post-menopausal patients above 70 were higher than those reported in studies with average age of post-menopausal patients below 70. This corroborates the increase of sclerostin level with age previously reported over the entire adult lifespan (Clarke and Drake, 2013). Similar to RANKL, only one study reported sclerostin in bone marrow plasma rather than serum (Mödder et al., 2011). The average sclerostin concentration reported in this study was within the range of concentrations reported by studies measuring serum sclerostin (Sarahrudi et al., 2012; Coulson et al., 2017).

Levels of TGF-β reported in the literature vary from 4.77 to 2,268 pM, respectively. The full range of concentrations reported in literature may be reduced to 77–220 pM using either the GRADE score or a differentiation based on demographics or measurement methods. TGF-β binds to and regulates differentiation of both osteoblast precursors and osteoclast precursors. Studies using ELISA assays designed for the detection of TGF-β1 only reported significantly different values relative to studies using methods designed for the detection of both TGF-β1 and TGF-β2. The data presented in Table 8 does not support a correlation between TGF-β concentration and age though this may be due to the fact that the demographics of participants in the studies summarized in Table 8 were similar.

### 3.2 Cell Numbers

In total, 22 peer-reviewed articles reporting measurements of osteoblast (N.Ob, *n* = 4), osteoclast (N.O_C_, *n* = 12), osteocyte (N.Ot, *n* = 6) numbers in post-menopausal women achieved a sufficient GRADE score for further analyses, the corresponding average cell numbers are listed in Tables 9–11, respectively. As with the cytokine concentrations, BMD, average patient age, N, origin of the sample and GRADE score were reported to better characterize the datasets prior to comparison. Additionally, measurement technique was reported to account for differences in the reported values. To facilitate comparison between reported cell numbers, any osteoclast or osteoblast numbers reported in the original article in units of cells/mm are reported here in both cells/mm and cells/mm^2^ and any osteocyte numbers reported in the original article in cells/mm^2^ are reported here in both cells/mm^2^ and cells/mm^3^. Cell numbers in different units were inferred from each other as outlined in Section 2.4.

**TABLE 9:** Osteoblast number measurements in postmenopausal women, in ascending order.

Study	n.ObL	BMD	Age	N	Sample origin	Measurement technique	Section Thickness	GRADE
HODSMAN 2000 (Hodsman et al., 2000)	0.59/mm ≈ 6.6/mm^2^	OP	65	15	Transiliac crest biopsy	Histology following ASBMR guidelines, 0.1% thionine staining, OsteoMeasure 2.2, OsteoMetrics, Decatur, GA, United States	5 µm	4
GRUBER 1986 (Gruber et al., 1986)	6.8/mm^2^	OP	62	14	Iliac crest biopsy, trabecular	Histology, identification of osteoblasts based on morphology, no staining, details of criteria used to identify osteoblasts not available	not available	2
GRUBER 2000 (Gruber et al., 2000)	1.0/mm ≈ 11/mm^2^	OP	64.5	18	Iliac crest biopsy	Histology, formalin/methacrylate sections, Goldner’s staining, osteoblasts identified as plump flattened or cuboidal cells that lined the osteoid surface	5 µm	2
jÄhn-rickert 2020 (Jähn-Rickert et al., 2020)	9.3/mm ≈ 100/mm^2^	all	65.5	43	Iliac crest biopsy, histology at central region of the cancellous bone compartment	Histology, Masson Goldner trichrome, toluidine blue and von Kossa/van Gieson staining, osteoblast numbers measured with toluidine blue but criteria for identification are not reported, OsteoMeasure, OsteoMetrics, Decatur, GA, United States	4 µm	5

Osteoblasts are the cells responsible for bone formation. Osteoblast numbers reported in literature vary between 6.6/mm^2^ and 100/mm^2^. The patient demographics for the four studies that fulfilled the inclusion criteria were very similar so there is no data to suggest a relationship between osteoblast numbers and age. The study with the highest GRADE score (5) reported the highest average osteoblast number and was also the most recent study and the only study to use toluidine blue staining (Jähn-Rickert et al., 2020). We propose that the bulk of the variation in reported average osteoblast numbers for similar patient demographics may be attributed to the different criteria used for the identification of osteoblasts. Gruber et al., 2000 used Goldner’s staining and then identified osteoblasts based on morphology as plump flattened or cuboidal cells that lined the osteoid surface while Jähn-Rickert et al., 2020, used toluidine blue staining and the quantitative histology software OsteoMeasure (OsteoMetrics, Decatur, GA, United States).

Average osteoblast numbers inferred in cells/mm^2^ from measurements in cells/mm were slightly higher than average osteoblast numbers reported in cells/mm^2^ in the original article, suggesting it may be necessary to develop a calibrated inference method. For all three studies in which inferences were made the osteoblast characteristic length was assumed to be 40 µm. Osteoblast characteristic lengths reported in literature vary between 20 and 50 µm (Qiu et al., 2019) so varying this parameter within the range reported in literature cannot explain the differences between osteoblast numbers reported in different units. One major limitation of the inference method is that it assumes a simplified trabecular geometry and may not be directly applicable to cortical bone. All articles found to report osteoblast numbers in postmenopausal women measured these numbers in iliac crest biopsies, which include a combination of cortical and trabecular bone. The biopsy technique will impact the proportion of cortical to trabecular bone.

Osteoclast numbers reported in literature vary between 0.06/mm^2^ and 2.35/mm^2^. The data presented in Table 10 includes studies with average participant ages between 50 and 72.8 and does not support a correlation between osteoclast number and age. Differentiating the studies according to the units in which osteoclast numbers were reported leads to two groups with similar average osteoclast numbers. Additionally, the data reported supports an association between the staining method and the measured average osteoclast numbers. Osteoclast numbers measured on histology sections stained with TRAP vary between 1.5 × 10^−1^/mm^2^ and 9.6 × 10^−1^/mm^2^ while osteoclast numbers measured on histology sections that were not stained or stained with any other staining method than TRAP (e.g. Goldner’s trichrome, toluidine blue, May-Grünwald-Giemsa, solochrome cyanin R) vary between 6 × 10^−2^/mm^2^ and 2.5 × 10^−1^/mm^2^. Furthermore, the GRADE score approach may be used to reduce the range of osteoclast numbers measured using TRAP staining to between 1.5 × 10^−1^/mm^2^ and 6.5 × 10^−1^/mm^2^ as the two largest osteoclast numbers reported in literature originated from low GRADE score studies.

**TABLE 10:** Osteoclast number measurements in postmenopausal women, in ascending order.

Study	N.OCL	BMD	Age	N	Sample origin	Measurement technique	Section Thickness	GRADE
CHavassieux 2013 (Chavassieux et al., 2013)	0.02/mm ≈ 0.06/mm^2^	OP	62	14	Transiliac bone biopsy	Paired biopsies, quantitative histology, staining with Goldner’s trichrome, solochrome cyanin R, toluidine blue, or May-Grünwald-Giemsa	8 µm	4
chavassieux 2019 (Chavassieux et al., 2019)	0.022/mm ≈ 0.066/mm^2^	OP	71.3	107	Transiliac biopsy	Histology, modified Goldner’s trichrome, solochrome cyanin R, toluidine blue, or May-Grünwald-Giemsa	8 µm	5
REHMAN 1994 (Rehman et al., 1994)	0.064/mm^2^ 0.0244/mm ≈ 0.073/mm^2^ 0.034/mm^2^	All	74	63	Trabecular Subcortical Cortical	Iliac crest biopsy, toluidine-blue stained sections	20 µm	3
RECKER 2020 (Recker et al., 2020)	0.09/mm^2^	all	72.8	40	Transiliac	Quantitative histology, Goldner and toluidine blue staining	5 µm	5
RECKER 2004 (Recker et al., 2004)	0.1/mm^2^	OP	66.44	14	Transiliac	Quantitative histology, Goldner trichrome staining	7.5 µm	5
WEINSTEIN 2009 (Weinstein et al., 2009)	0.05/mm ≈ 0.15/mm^2^	OP	50	55	Iliac crest biopsy	Histology with TRAP staining, osteoclasts 80 to >100 µm thickness	5 µm	3
CHAVASSIEUX 1997 (Chavassieux et al., 1997)	0.057/mm ≈ 0.17/mm^2^	OPE	44–84	31	Transiliac bone biopsy	Goldner staining, Semiautomatic image analyzer Ibas 1 Leica, Wetzlar, Germany Automatic image analyser Visiolab 5,000 Biocom, Les Ulis, France	not available	3
carasco 1989 (Carasco et al., 1989)	0.28/mm^2^ 0.22/mm^2^	non-OP OP	62.5	69	Iliac crest biopsy	Quantitative histology, Leitz analyzer	5 µm	4
GRUBER 2000 (Gruber et al., 2000)	0.07/mm ≈ 0.21/mm^2^	OP	62	14	Iliac crest biopsy	Histology, Goldner’s staining, osteoclasts identified as multinucleated cells in direct contact with the endosteal surface	5 µm	2
jähn-rickert 2020 (Jähn-Rickert et al., 2020)	0.07/mm ≈ 0.21/mm^2^	all	65.5	43	Iliac crest biopsy	Quantitative histology with TRAP staining	4 µm	5
DEMPSTER 2018 (Dempster et al., 2018) REID 2010 (Reid et al., 2010)	0.08/mm ≈ 0.24/mm^2^	OP	63.45	69	Iliac crest biopsy	Quantitative histology with TRAP staining	7 µm	4
Arlot 1990 (Arlot et al., 1990)	0.03/mm ≈ 0.09/mm^2^ 0.14/mm ≈ 0.42/mm^2^	non-OP OP	66	63	Transiliac biopsy	Quantitative histology, endocortical and cancellous, Goldner staining, morphological identification	7 µm	4
HODSMAN 2000 (Hodsman et al., 2000)	0.13/mm ≈ 0.39/mm^2^	OP	65	15	Transiliac crest biopsy	Quantitative histology, ASBMR guidelines, 0.1% thionine staining, OsteoMeasure 2.2, OsteoMetrics, Decatur, GA, United States	5 µm	4
Ott 2009 (Ott et al., 2009)	0.65/mm^2^	OP	68	65	Transverse biopsy anterior iliac crest	Quantitative histology, Goldner and TRAP stained, OsteoMeasure, OsteoMetrics, Decatur, GA, United States	5–8 μm	5
JOBKE 2014 (Jobke et al., 2014)	0.22/mm ≈ 0.66/mm^2^	All	52.6	23	Dorsal iliac crest biopsy	Quantitative histology with TRAP staining	5 µm	2
GRUBER 1986 (Gruber et al., 1986)	0.96/mm^2^	OP	62	14	Iliac crest biopsy	Quantitative histology with TRAP staining	not available	2
Dekker 2018 (Dekker et al., 2018)	0.364/mm ≈ 1.09/mm^2^ 0.396/mm ≈ 1.18/mm^2^	non-OP	65	9 17	Cort. mandible Trab. mandible	Quantitative histology with Goldner and TRAP staining	5 µm	2
COHEN-SOLAL 1995 (Cohen-Solal et al., 1995)	2.35/mm^2^	non-OP	66	16	Cancelllous femoral neck biopsy	Semiautomatic image analyzer, Biocom, Les Ulis, France	7 µm	2

Osteocyte numbers reported in literature vary between 1.56 × 10^4^/mm^3^ and 3.09 × 10^4^/mm^3^. The measurement location and in particular the differentiation between trabecular and cortical bone is a key element that may be used to refine the estimate of the osteocyte number. The data collected in Table 11 corroborates previously reported conclusions that the osteocyte density is higher in cortical than in trabecular bone (Akhter et al., 2017). All three studies measuring osteocyte numbers in both osteoporotic and non-osteoporotic patients report lower osteocyte numbers in osteoporotic patients.

**TABLE 11:** Osteocyte number measurements in postmenopausal women, in ascending order.

Study	N.OT	BMD	Age	N	Sample origin	Measurement technique	Section Thickness	GRADE
QIU 2010 (Qiu et al., 2010)	148/mm^2^ ≈ 18,500/mm^3^	non-OP	52.9	8	Iliac bone biopsies, trabecular	bright-field light microscope (×20 objective) equipped with a Bioquant Image Analysis System (Bioquant Nova, Nashville, TN, United States) Goldner’s trichrome staining, to differentiate occupied vs. empty lacunae	5 µm	4
Qiu 2003 (Qiu et al., 2003)	188/mm^2^ ≈ 23,500/mm^3^ 125/mm^2^ ≈ 15,625/mm^3^	non-OPE OP	62.2 66.2	100	Transiliac bone biopsy	Optical (bright field light) microscope (R&M Biometrics Inc.), Goldner’s trichrome staining, occupied lacunae defined as the stained lacunae	5 µm	4
Rolvien 2020 (Rolvien et al., 2020)	158.5/mm^2^ ≈ 19,813/mm^3^ 137.6/mm^2^ ≈ 17,200/mm^3^	non-OP OP	81 80	9 11	Cadaver femur	Scanning electron microscope (LEO435 VP), Frontier FTIR spectrometer (Universal ATR, MA)	5 µm	3
Dong 2014 (Dong et al., 2014)	20,573/mm^3^	All	79	2	Cadaver human cortical femoral mid-diaphysis	European Synchrotron Radiation Facility, Grenoble, France, beamline ID19, total angle 360° fixed energy 25 keV, resolution 1.4 µm	NA	5
akhter 2017 (Akhter et al., 2017)	20,850/mm^3^	All	53.9	8	Transiliac bone biopsy	MicroXCT-200 (Carl Zeiss X-Ray Microscopy, Pleasanton, CA)	NA	2
mullender 2005 (Mullender et al., 2005)	271.3/mm^2^ ≈ 33,913/mm^3^ 222.6/mm^2^ ≈ 27,825/mm^3^	non-OPE OP	64.4 69.6	53	Transiliac bone biopsy	bright-field light microscope with TAS + image analyzer TAS + by Leitz, Wetzlar, Germany ×25 magnification	7 µm	4
Milovanovic 2014 (Milovanovic et al., 2014)	247/mm^2^ ≈ 30,875/mm^3^	OP	82	4	Transiliac bone biopsy, cortical	resolution 10 μm, μCT 40, Scanco Medical, Brüttisellen, Switzerland	NA	3

### 3.3 *In Vitro* and *In Silico* Reports to Fill Gaps in the Clinical Literature

Although the parameters summarized up to now form the basis for the initialization of micro-MPA models of bone or bone cell population dynamics models, other parameters are needed as well to accurately represent the complexity of the pathways responsible for bone remodeling. For cytokine dynamics, properties such as diffusion rate, rate of decay and the rate of production by bone cells are required. Regarding cell numbers, the rates of cell proliferation, apoptosis, differentiation, motility and reaction rates as well as binding site occupancy are needed. The proposed search strategy did not lead to the identification of any articles measuring these rates directly in postmenopausal women. As such, the authors suggest an alternative strategy to reduce the parameter range using a system inspired by the levels of evidence of the Oxford Center for Evidence-based Medicine, developed for therapeutic decision making (Howick et al., 2011).

Table 12 provides an overview of the proposed method to assess the quality of the evidence for the measure reported for each parameter necessary for the setup of a model of bone remodeling incorporating cell and cytokine dynamics. Ideally, cell and cytokine behavior parameters should be directly measured *in vivo* in the patient population of interest, i.e. postmenopausal women for the purpose of this review. The search strategy adopted in this review did not identify any such measurements and to the best of our knowledge there are to date no published reports of intravital cell imaging in humans. To reduce the parameter space, alternatives include measuring cell and cytokine behavior parameters in conditions resembling *in vivo* conditions by extracting a biopsy or bone marrow supernatant fluid, isolating the cells and culturing them in an environment resembling bone (evidence level 1 in Table 12) or to perform intravital cell imaging in animals (evidence level 2 in Table 12). However, cells are most often cultured in Petri dishes or other 2D environments that are not representative of bone (evidence level 3 in Table 12). Cell types studied in such environments may not be cells isolated from humans but rather analog cell strains designed specifically for cell culture (evidence level 4 in Table 12). Lastly, computational studies may shed light on the range of values that can theoretically be expected to generate simulations whose trabecular morphometrics resemble *in vivo* measurements (evidence level 5 in Table 12).

**TABLE 12:** Levels of Evidence approach to reduce the parameter range for missing parameters in micro-MPA models or bone cell population dynamics models of osteoporosis and its treatments in postmenopausal women. Inspired by the Levels of Evidence approach of the Oxford Centre for Evidence-Based Medicine (Levels of Evidence Working Group, 2011).

		LEvel 1	Level 2	Level 3	Level 4	Level 5
Parameter of interest	Cell proliferation, differentiation, motility, apoptosis rate or lifespan	*Ex vivo* live cell imaging of cells extracted directly from a population of post-menopausal women (optimally longitudinal paired biopsies). Characterization in biopsy itself or in a mechanical and chemical environment mimicking *in vivo* (cyclic loading, fluid flow, bone organoids … )	*In vivo* live cell imaging in animals. Ovariectomy to represent menopause. Immunohistochemistry for local cell analysis	*In vitro* culture of cells extracted from a population of non-female/non-elderly patients. Cell culture in 2D in a mechanical and chemical environment that does not attempt to replicate *in vivo* conditions	*In vitro* characterization of cell behavior for non-human cell strains designed for cell culture or animal cells (murine or tea) Cell culture in 2D in a mechanical and chemical environment that does not attempt to replicate *in vivo* conditions	*In silico* study reporting trends in morphometrics or other parameter using a given parameter estimate/unreasonable results with another parameter. Unpublished/self-designed *in silico* study. Back-of-the envelope calculation based on cell populations in longitudinal histology slices
	Cell-cytokine and cytokine-cytokine binding and impact of binding site occupancy on cell behavior	Cell behavior measured as above. Cytokine concentrations evaluated in BMSF. Repeated measurements, spatial and temporal random sampling. Validated commercial ELISAs and binding models	Cell behavior measured as above. Cytokine concentrations evaluated in animal BMSF. Validated commercial animal ELISAs and binding models	Cell behavior measured as above. Cytokine concentrations also *in vitro*, theoretical models for cell-cytokine binding e.g. Hill-Langmuir equation and cytokine-cytokine binding, e.g. competitive reaction equilibrium equations	Cell behavior measured as above. Cytokine concentrations also *in vitro*, theoretical models for cell-cytokine binding e.g. Hill-Langmuir equation and cytokine-cytokine binding, e.g. competitive reaction equilibrium equations	Indirect derivation based on computational studies or theoretical models for cell-cytokine binding e.g. Hill-Langmuir equation and cytokine-cytokine binding, e.g. competitive reaction equilibrium equations
	Cytokine production rates by cells and cytokine decay constants and diffusivity	Gene expression studies to quantify cytokine production directly in postmenopausal women, BMSF cytokine measurements	Longitudinal and spatially distributed cytokine assays in serum or animal BMSF	As in the first row but coupled with cytokine assays in serum	Cytokine concentrations also *in vitro*, theoretical models for cell-cytokine binding e.g. Hill-Langmuir equation and cytokine-cytokine binding, e.g. competitive reaction equilibrium equations	Cytokine production rates by cells that lead to *in silico* or mathematical results consistent with *in vivo* measurements
Number of participants		>5	>5	>5	≤5	>5

The proposed levels of confidence approach involves searching in the literature first for cell behavior parameters at evidence level 1, i.e. for human cells measured in an environment resembling that *in vivo*. Then, in the absence of any such data, investigating data with a lower confidence level and so on until evidence level 5 is reached. An appropriate illustration of this process for the determination of the apoptosis rate or lifespan of osteoclasts is as follows:

Conflicting evidence suggests osteoclasts have a lifespan from 2 weeks (Manolagas, 2000) up to 6 months (Jacome-Galarza et al., 2019; Søe, 2020), with the differences in those values reconciled at least in part by the discovery of osteomorphs (McDonald et al., 2021); this cell type is generated by the fission of osteoclasts into daughter cells as an alternative to apoptosis thereby increasing the cell lifespan. Both the osteoclast lifespan extremes, i.e. 2 weeks and 6 months, were measured *in vitro* in murine osteoclasts (Hughes et al., 1996; Jacome-Galarza et al., 2019) (evidence level 4). Fission and fusion into osteomorphs as an alternate cell fate to apoptosis (McDonald et al., 2021) was measured using intravital imaging in mice (evidence level 2) and *in vitro* culture of human osteoclasts (evidence level 3). Past *in silico* studies may have justified the use of any osteoclast lifespan within the denoted range to setup their model. Our recommendation moving forward is for conflicting evidence to be assessed according to the levels presented in Table 12 and only the top level of evidence used to parametrize future models.

Note that the level of evidence for parameters governing ligand-binding site kinetics is assessed slightly differently from that for cell behavior parameters (Table 12). Direct *in vivo* measurements of ligand-binding site kinetics were not found with the search strategy used in this review but are essential for the regulation of cellular behavior in micro-MPA models of bone mechanobiology. The absence of evidence of level 1 through 3 implies that the only way to estimate the ligand-binding site kinetics of interest is relying on level 4 and level 5 evidence. Level 4 evidence in this context includes reaction rates and binding site occupancy estimated from *in vitro* studies, e.g. 6,382 to 8,491 LRP6 receptors per cell (Lee et al., 2018). Level 5 evidence includes peer-reviewed computational studies, self-designed unpublished computational studies, and back of the envelope calculations with the level of confidence in the point estimates decreasing along that list. Examples of level 5 evidence include reports of 1000 TGF-β receptors/cell (Schafer et al., 2013), a maximum number of 2.703 × 10^6^ RANK receptors per osteoblast (Scheiner et al., 2013) and a maximum number of 3 × 10^6^ RANK receptors per osteoblast (Pivonka et al., 2008).

Finally, the proposed strategy to assess the level of evidence for cytokine production levels by bone cells and cytokine decay and diffusivity is presented in row 3 of Table 12. Evidence of level 1 for these parameters includes both gene expression studies in bone cell cultures derived from bone biopsies in postmenopausal women and longitudinal measurements of cytokine concentrations. ELISA measurements reporting 0.62 ± 0.23 ng of TGF-β1 stored per mg of mineralized bone in iliac crest biopsies from postmenopausal women are an example of level 1 evidence in this context (Bismar et al., 1999). This measurement may be used in an micro-MPA model to define the amount of TGF-β1 released when the bone matrix is resorbed by osteoclasts. Evidence of level 2 includes live cell imaging in animals coupled with genomics studies; however, to the best of our knowledge, there are no examples of this type of evidence for cytokine production rates within the literature. Evidence of level 3 involves live cell imaging in 2D cell cultures of human cells and genomic analyses and longitudinal cytokine assays of these *in vitro* cultures. Most of the evidence relating to cytokine production levels by bone cells may be categorized as level 3. As such, parameters may be derived indirectly from calculations based on measurements of cytokine concentrations and cell numbers (level 5) (Table 12). As an illustrative example, TGF-β production can be derived in such fashion as it is released from the bone matrix as a result of the activity of only 1 cell type, osteoclasts. One level 5 approach to reduce the parameter range for the amount of TGF-β released per active osteoclast is to divide the total change in TGF-β concentration in conditions of high stimulation such as fracture healing by the time and the osteoclast number.

Level 5 evidence may involve indirect derivations of the measures of interest based on other measures, as illustrated by the above estimate of TGF-β production levels. Features of individual cells and/or cellular units can be used for indirect verification. The functional unit of cells in bone, referred to as the basic multicellular unit (BMU), composed of osteoclasts followed by osteoblasts, has been reported to measure approximately 2 mm end to end (Parfitt, 1994). Further verification elements may come from lifespans reported for BMUs (e.g., 6–9 months (Manolagas, 2000)), stem cells in general (e.g., 5 months to 3 years (Sieburg et al., 2011)) and information concerning the make-up of the activation-resorption-formation cycle (e.g., osteoclasts start resorbing bone as a team of 10–20 cells (Manolagas, 2000)). Moreover, MSCs cannot proliferate beyond the Hayflick limit of approximately 50 population doublings and their long axis has a size of approximately 200 microns (Charbord, 2010).

The examples provided above are by no means exhaustive. The choice of parameters to include will vary from one micro-MPA model to the next; therefore, a comprehensive overview of the *ex vivo*, *in vitro* and *in silico* literature that may be used as tools to reduce the parameter range is outside the scope of this review. In addition, for a number of these parameters there is no evidence at any level, be it 1, 2, 3, 4 or 5, available in the literature. In these cases, we propose that the possible range for parameters of interest for micro-MPA models may be refined by running micro-MPA model simulations with a broad range of values for the missing parameters and comparing the densitometric, static morphometric and dynamic morphometric characteristics of the simulation output with *in vivo* measurements reported in clinical trials of treatments for osteoporosis to determine the range of settings giving stable physiologic output.

## 4 Limitations

Limitations of this review include the fact that the authors only considered parameters that have to date been included in published *in silico* micro-MPA models of OP and its treatments. Although other molecules and cell types have been hypothesized to play significant roles in bone remodeling, the authors chose to focus the review on existing model parameters as these are most likely to be implemented in future models. The authors acknowledge the off-label use of the GRADE approach, which is traditionally used as a framework for developing and presenting summaries of evidence for clinical practice recommendations. However, adaptations to the traditional approach were described in a detailed manner to ensure transparency and applied consistently across individual studies to assess the data presented in this study. Note that the adapted GRADE system described in this review may be implemented to develop other parameter sets for other diseases, but this may require that the data reporting become more standardized. A shortcoming to the confidence in the point estimates of the data included in this review would be the error introduced due to the assumptions made during the conversion of units of data as given in the published sources from the standard units presented in this review. To remove ambiguity we have mentioned the conversion factors whenever the data was transformed.

## 5 Conclusion

This systematic review has identified a collection of *in vivo* data to assist in the parametrization of cell and cytokine behavior in micro-MPA models and bone cell population dynamics models. For some of these parameters, we have highlighted criteria and methods that may be used to reconcile differences between measurements reported in various articles. Default levels of RANKL can be set for patients with and without OP. Sclerostin and estrogen should be adjusted by patient age. Measured osteoblast and osteoclast numbers may be affected by measurement methodology and in particular the staining procedure or morphological criteria used to identify the cell type of interest. Osteocyte numbers should be scaled to the disease state. Additionally, the selection of literature parameters to set up *in silico* models of bone may be facilitated by comparing the quality of conflicting evidence using an adaptation of the GRADE system. Outliers in the reported cell numbers, cytokine concentrations and even densitometric and morphometric trends were exclusively low GRADE score studies and in this respect the proposed adapted GRADE system proved to be a viable assessment method for modelers to evaluate the reliability of parameters reported in clinical studies. We have outlined how, by eliminating low GRADE score studies, the parameter range for cell numbers and cytokine concentrations may be reduced, while also defining the level of confidence in this parameter range reduction. The conclusions made here are based on published work and established methods, however with time and advances in measurement technique, the parameter range for each cell and cytokine behavior setting or predictive target will vary and be refined for more specific patient demographics, diseases and treatments. Thus, a living inventory is available as an open access resource here.

This review clarified the scarcity of clinical data on reaction rates, binding site occupancy, cytokine diffusion and decay and cytokine production levels by bone cells. Gaps in the clinical evidence may be filled with human *ex vivo*, animal *in vivo*, human *in vitro*, non-human *in vitro* or *in silico* evidence. To assess the reliability of point estimates used to fill gaps in the literature, we propose an adaptation of the Oxford CEBM Levels of Evidence approach. This approach can be used to determine the order of magnitude of micro-MPA model parameters for which there is no clinical evidence. Future studies should use this method and report the level of evidence for cell and cytokine behaviour parameters used in the parametrization of their model. The methods outlined in the review can be applied to other clinical trial literature and metabolic bone diseases, opening the door to better standardization across the field and a scientific consensus on quantitative characteristics of bone cell and cytokine behaviour and the identification of targets for further *in silico* research.

## Data Availability

The datasets presented in this study can be found in online repositories. The names of the repository/repositories and accession number(s) can be found below: 10.3929/ethz-b-000548897.
