# Supplementary material for: Clinical Data for Parametrization of In Silico Bone Models Incorporating Cell-Cytokine Dynamics: A Systematic Review of Literature
Source: Front Bioeng Biotechnol. 2022 Jul 12;10:901720. doi: 10.3389/fbioe.2022.901720 (PMC9335409; doi:10.3389/fbioe.2022.901720)
Supplement: Supplementary file 1 [file DataSheet2.pdf]

Exclusion criteria

Scientific articles identified during the search process were excluded in all the following cases:

The article was a review, book chapter, or report of *in silico* or *in vitro* research. Note that *ex vivo* measurements were included.
The article reported data for whole body vibration or physical activity including resistance exercise, pulsed electromagnetic fields or long term immobilization
The article reported data for individuals with a different disease (e.g. thalassemia, bone marrow oedema, chronic kidney disease (CKD) with haemodialysis, renal osteodystrophy, glucocorticoid-induced PMO, osteoporosis in young people, cerebral palsy, hyperthyroidism, hypothyroidism, psoriasis, osteomalacia, osteoarthritis, osteogenesis imperfecta, Kummell’s disease, sarcopenia, pedicle screw fixation, hypophosphatasia, melorheostosis, HIV, breast cancer, etc.)
The article reported data for a different subject population (e.g. rats, rabbits, monkeys, dogs, sheep, astronauts from the international space station (ISS), male only, young women only, women with HIV, only patients with autografts, obese women, patients with volar locking plate fixation, only women with fractures with assessment at fracture site, premenopausal, pregnant or lactating women, spinal fusion surgery, dental implant, only peri-implant bone percutaneous vertebroplasty, etc.)
The article included non-approved PMO treatments (e.g. etidronate, odanacatib, aromatase inhibition, vitamin K, blosozumab, etidronate, clodronate, pamidronate, tocotrienol, astaxanthin, Qing’e formula, dried plum, soyisoflavone or carotenoid)
The article reported measures at different sites or different measures (e.g. jaw, studies on effects of gene polymorphisms without measurements of cytokine concentrations, coronary tortuosity, HR-pqCT of muscle and/or myotendinous tissue, FGF-23, persistence, miRNAs, quantitative ultrasound or breast cancer incidence)
