# Supplementary material for: Clinical Data for Parametrization of In Silico Bone Models Incorporating Cell-Cytokine Dynamics: A Systematic Review of Literature
Source: Front Bioeng Biotechnol. 2022 Jul 12;10:901720. doi: 10.3389/fbioe.2022.901720 (PMC9335409; doi:10.3389/fbioe.2022.901720)
Supplement: Supplementary file 2 [file DataSheet3.pdf]

Remarks on postprocessing of data from literature

Please refer to our open source living inventory at https://doi.org/10.3929/ethz-b-000548897 for detailed insights on the post-processing of literature values in this review. A few points may be worthy of emphasis here:

In articles in which baseline measurements of cell numbers or cytokine concentrations as well as placebo measurements at several times were available, we chose to report only the average of the values reported weighted by the number of postmenopausal women in which each measurement was performed. An example of this is the data from Chavassieux 1997 [1]. At the 24-month timepoint, an average osteoclast number of 0.082/mm was measured in 31 patients receiving placebo and at the 36 months timepoint an average osteoclast number of 0.038/mm was measured in 40 patients receiving placebo. This information was concisely summarized as an average osteoclast number of (0.082 x 31 + 0.038 x 40) /(31 +40) = 0.057/mm in 71 patients. Similarly, when data was reported for several subdemographics within the category postmenopausal women (for example stratified according to age), the data was summarized by reporting only an average of the parameter of interest weighted by patient population. As an example, in [2] average osteoclast numbers were measured to be 2.1/mm in 30 women in their sixties, 2.2/mm in 17 women in their seventies, 3.5/mm in 10 women in their eighties, 3.1/mm in 6 women in their nineties. This was summarized as (2.1 x 30 + 2.2 x 17 + 3.5 x 10 + 3.1 x 6) / (30 + 17 + 10 + 6) =2.44/mm.

This review aimed to compare and reconcile the various values reported in literature for parameters relevant for the setup of micro-multiphysics agent-based models of bone. For this purpose it was key to select comparable measures so for example articles reporting cell surfaces rather than cell number[3,4] were excluded as there is no convention to infer cell surfaces from cell number.

As detailed in section 2.4. Methodology for Analysis of Physiologic Ranges for Cell and Cytokine Parameters, to facilitate comparison between cell numbers reported in various units, we proposed a simple inference method consisting of dividing cell numbers in cells/mm by (2.23 x cell characteristic length) to obtain cells/mm^2. The factor of 2.23 was included to account for surface curvature and the characteristic length of osteoblasts was defined as 40 μm and that of osteoclasts as 150 μm. In a few rare cases, studies reported both cells/mm and cells/mm^2 thus providing some form of validation dataset for this inference method. The data in these studies (e.g. Rehman et al. 1994 [2]) is consistent with our inference method, as shown in greater detail in our online inventory.

1 Chavassieux, P. M. *et al.* Histomorphometric assessment of the long-term effects of alendronate on bone quality and remodeling in patients with osteoporosis. *J. Clin. Invest.* **100**, 1475-1480, doi:10.1172/JCI119668 (1997).

2 Rehman, M. T., Hoyland, J. A., Denton, J. & Freemont, A. J. Age related histomorphometric changes in bone in normal British men and women. *J. Clin. Pathol.* **47**, 529-534, doi:10.1136/JCP.47.6.529 (1994).

3 Eriksen EF, Melsen F, Sod E, Barton I, Chines A. Effects of long-term risedronate on bone quality and bone turnover in women with postmenopausal osteoporosis. Bone. 2002 Nov;31(5):620-5. doi: 10.1016/s8756-3282(02)00869-4. PMID: 12477578

4 Cohen-Solal ME, Graulet AM, Gueris J, Denne MA, Bergot C, Morieux C, Sedel L, Kuntz D, De Vernejoul MC. Bone resorption at the femoral neck is dependent on local factors in nonosteoporotic late postmenopausal women: an in vitro-in vivo study. J Bone Miner Res. 1995 Feb;10(2):307-14. doi: 10.1002/jbmr.5650100219. PMID: 7754812.
