# Supplementary material for: Clinical Data for Parametrization of In Silico Bone Models Incorporating Cell-Cytokine Dynamics: A Systematic Review of Literature
Source: Front Bioeng Biotechnol. 2022 Jul 12;10:901720. doi: 10.3389/fbioe.2022.901720 (PMC9335409; doi:10.3389/fbioe.2022.901720)
Supplement: Supplementary file 3 [file DataSheet1.pdf]

List of Abbreviations, Acronyms and Terminology

	Abbreviation	Definition
Evidence assessment techniques, diseases and modelling techniques	GRADE	Grading of Recommendations, Assessment, Development and Evaluations
	CEBM	Centre for Evidence-Based Medicine
	OP	Osteoporosis
	PMO	Post-Menopausal Osteoporosis
	RVE	Representative Volume Element
	micro-MPA	micro-MultiPhysics Agent-based
Cytokines	TGF-β	Transforming Growth Factor β
	RANKL	Receptor Activator of Nuclear Factor κ β Ligand
	OPG	Osteoprotegerin
	PTH	ParaThyroid Hormone
	SCLR	sclerostin
	BMP	Bone Morphogenetic Protein
	TNF	Tumour Necrosis Factor
	Wnt	Wingless and int-1
	IGF	insulin-like growth factor
	MCSF	macrophage colony stimulating factor
	NO	nitric oxide
	Sema3A	semaphorin 3A
	RUNX2	Runt-related transcription factor 2
Cells	N.Oc	Osteoclast number

	N.Ob	Osteoblast number
	N.Ot	Osteocyte number
	MSCs	Mesenchymal Stem Cells
	HSCs	Hematopoietic Stem Cells
	BMU	Basic Multicellular Unit
Measurement techniques and related terms	CT	Computed Tomography
	micro-CT	micro-scale Computed Tomography
	DXA	Dual-energy X-ray Absorptiometry
	BMD	Bone Mineral Density
	TRAP	Tartrate Resistant Acid Phosphatase
	LC-MS	Liquid Chromatography with tandem Mass Spectrometry
	ELISA	Enzyme-Linked ImmunoSorbent Assay
	RIA	RadioImmunoAssay
	CV	Coefficient of Variation
	FTIR	Fourier Transform Infrared Spectroscopy
	BMSF	Bone Marrow Supernatant Fluid
